# Supplementary material for: Subventricular zone cytogenesis provides trophic support for neural repair in a mouse model of stroke
Source: Nat Commun. 2023 Oct 10;14:6341. doi: 10.1038/s41467-023-42138-0 (PMC10564905; doi:10.1038/s41467-023-42138-0)
Supplement: Supplementary file 1 — Supplementary Information [file 41467_2023_42138_MOESM1_ESM.pdf]

Supplementary Information

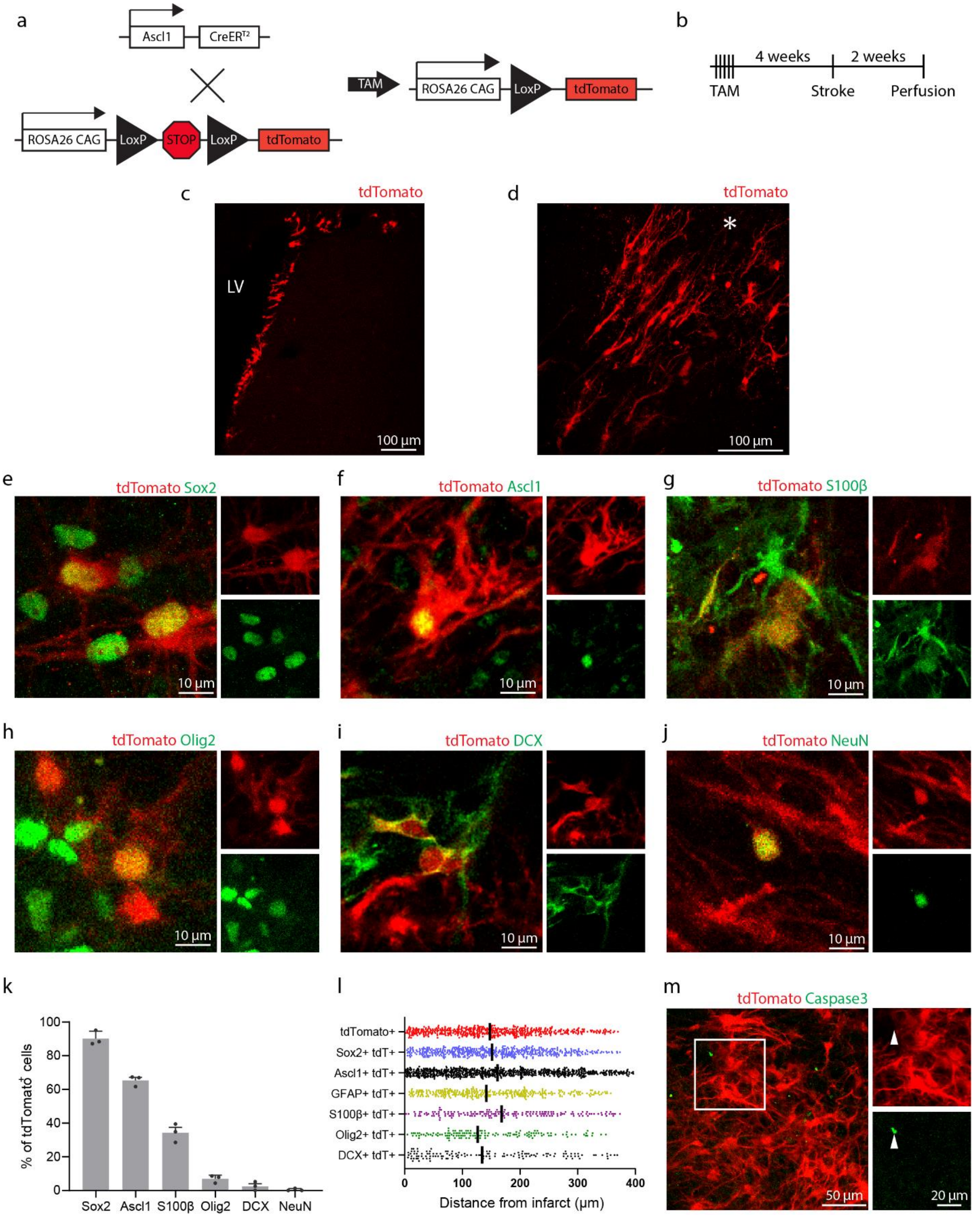

Supplementary Fig. 1. SVZ-derived cells lineage traced with *Ascl1^{CreERT2}* are predominantly undifferentiated precursors and astrocytes.

a) Schematic of genetic lineage tracing system where tamoxifen (TAM) induces indelible expression of tdTomato in Ascl1-expressing neural stem cells, intermediate progenitor cells, and their progeny. **b)** Experimental design. **c)** Image showing tdTomato expression in the SVZ. LV, lateral ventricle. **d)** Representative image of tdTomato^{+} cells localized to peri-infarct cortex 2 weeks after a cortical stroke (asterisk). **e-j)** Lineage tracing using *Ascl1^{CreERT2}*; Ai14 mice corroborates the major finding of Figure 1, that SVZ-derived cells in peri-infarct cortex are predominantly undifferentiated precursors and astrocytes. Representative images (n = 3 mice) illustrating co-labeling of lineage traced tdTomato^{+} cells with differentiation stage-specific markers at two weeks post-stroke **(e)** Sox2, **f)** Ascl1, **g)** S100β, **h)** Olig2, **i)** DCX. **j)** NeuN). **k)** Quantification of marker expression by % of tdTomato^{+} cells. n = 3 mice. Data are presented as mean ± SEM. **l)** Spatial distribution of cells relative to the infarct border by marker expression at 2 weeks post-stroke in *Nestin^{CreERT2}*; Ai14 mice. Each point indicates a single cell. Vertical lines indicate medians. There was no clear pattern of spatial organization by cell type. Distance was measured as the distance to the nearest part of the infarct border within a given section for each cell. Only cells within 400 µm of the infarct border and within cortex were included in this analysis. n = 3 mice. **m)** No evidence for cleaved caspase 3 expression in SVZ-derived cells localized to peri-infarct cortex. 0 of 437 tdTomato^{+} cells counted expressed cleaved caspase 3 (n = 3 *Nestin^{CreERT2}*; Ai14 mice at 2 weeks post-stroke). Arrowhead indicates a cleaved caspase 3^{+} tdTomato^{-} cell. All images are representative of 3 mice. Source data are provided as a Source Data file.

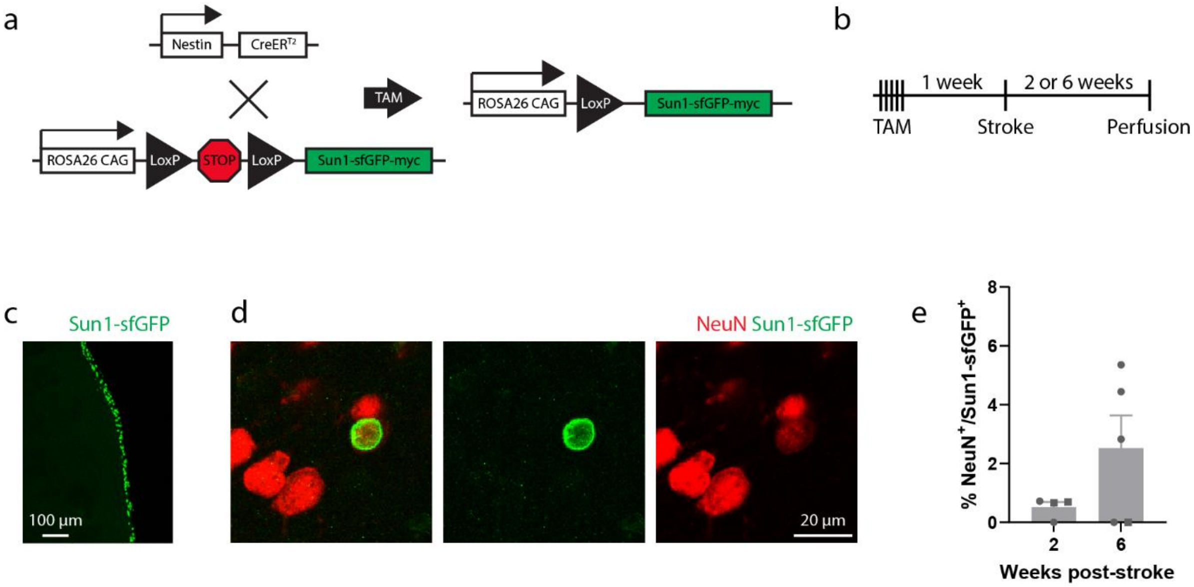

Supplementary Fig. 2. Few SVZ-derived cells become neurons.

a) Schematic of genetic lineage tracing system where tamoxifen (TAM) induces indelible expression of a nuclear membrane-bound Sun1-sfGFP in neural stem cells and their progeny. **b)** Experimental design. **c)** Image showing Sun1-sfGFP expression in the SVZ. Image is representative of 9 mice. **d)** Representative image of a $NeuN^{+}Sun1\text{-}sfGFP^{+}$ nucleus in peri-infarct cortex. **e)** Quantification of SVZ-derived neurons. Data were derived from a combined 1014 nuclei counted across nine mice (n = 4 mice at two weeks, n = 5 mice at six weeks). There was not a significant difference between time points ($t(4.2) = 1.79$, $p = 0.145$, Welch's corrected two-tailed t test). Data are presented as mean ± SEM. Datapoints representing males are shown as circles; datapoints representing females are shown as squares. Source data are provided as a Source Data file.

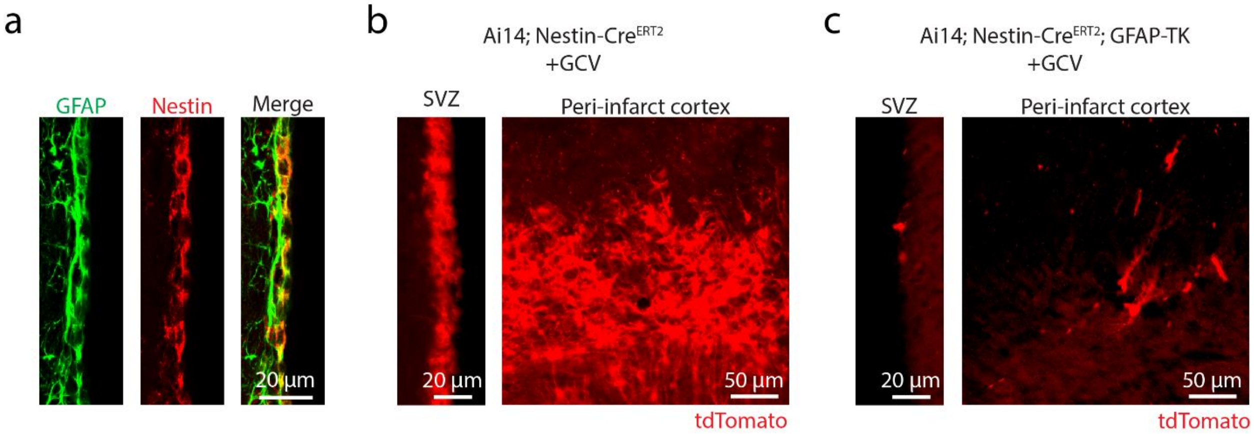

Supplementary Fig. 3. Overlap of Nestin$^+$ and GFAP$^+$ cells in the SVZ.
a) Confocal image showing overlap of GFAP and Nestin in the SVZ. Nestin$^+$ cells are also GFAP$^+$. Nestin$^-$ GFAP$^+$ cells are most likely differentiated astrocytes. **b, c)** Fluorescent images of tdTomato$^+$ cells in the SVZ and peri-infarct cortex of mice two weeks after stroke. Ai14; Nestin-Cre^{ERT2} (b) and Ai14; Nestin-Cre^{ERT2}; GFAP-TK (c) mice were treated with ganciclovir (GCV) for two weeks prior to stroke. During the last five days of GCV treatment mice were injected with tamoxifen daily to induce tdTomato expression in Nestin-expressing cells. There was a substantial loss of tdTomato$^+$ cells in mice with the GFAP-TK transgene, demonstrating that Nestin-Cre^{ERT2} and GFAP-TK mouse lines target a largely overlapping population of SVZ precursors. All images are representative of experiments repeated in 3 mice.

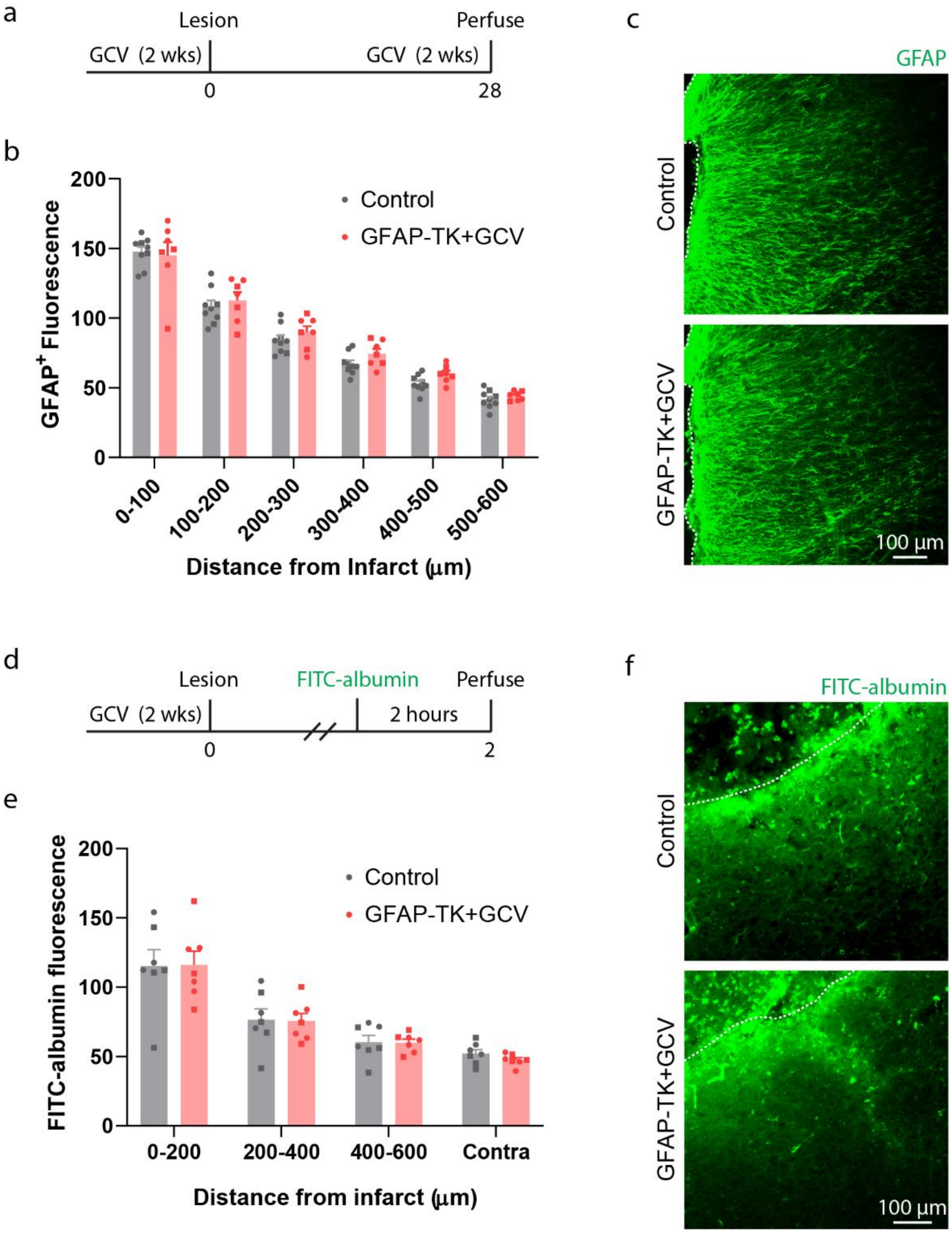

Supplementary Fig. 4. Neural stem cell ablation does not alter parenchymal astrocyte reactivity or vascular permeability after stroke.

a) Experimental timeline for examining GFAP fluorescence. Tissue was obtained 28 days post-stroke. **b)** $GFAP^+$ fluorescence was increased near the lesion, but declined with distance away, characteristic of astrocyte reactivity (two-way ANOVA, distance effect $F(5, 84) = 172.9$, $p < 0.0001$). There was no significant group effect ($F(1, 84) = 2.8$, $p = 0.100$). n = 9 controls ($TK^{-/-}$), n = 7 GFAP-$TK^{+/-}$. **c)** Representative images of GFAP immunostaining in peri-infarct cortex.

Dashed lines indicate the lesion border. **d)** Experimental design for examining vascular permeability. Two days post-stroke, animals were retro-orbitally injected with FITC-conjugated albumin 2 hours before perfusion. **e)** FITC-albumin fluorescence was greatest near the infarct border and declined with distance away, consistent with injury-induced vascular permeability (Two-way ANOVA, distance effect $F(3, 48) = 36.9$, $p < 0.001$). There was no significant effect of group ($F(1, 48) = 0.07$, $p = 0.789$). n = 7 controls ($TK^{-/-}$), n = 7 GFAP-$TK^{+/-}$. **f)** Representative images of FITC-albumin fluorescence in peri-infarct cortex. Dashed lines indicate the lesion border. Data are presented as mean ± SEM. Datapoints representing males are shown as circles; datapoints representing females are shown as squares. Source data are provided as a Source Data file.

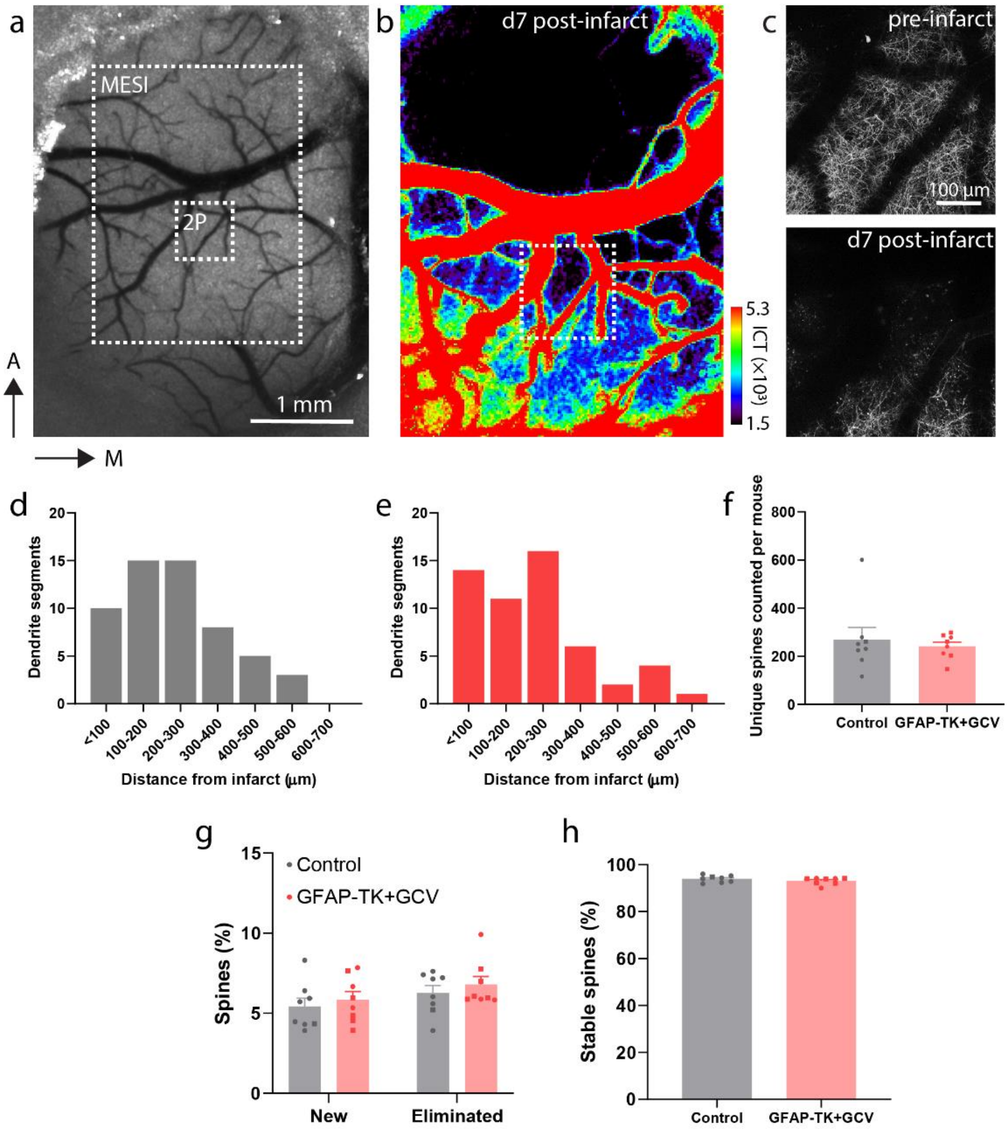

Supplementary Fig. 5. Additional longitudinal imaging data.
a) MESI accurately delineates the infarct border. Widefield laser speckle contrast image showing the cortical surface through a cranial window. **b)** Multi-exposure speckle imaging (MESI) image of blood flow corresponding to the region indicated in panel a. **c)** Two photon image of GFP-labeled apical dendrites (Thy1-GFP) corresponding to the region indicated in panels a and b. The infarct border revealed by MESI (black region in b) matches the region in which GFP fluorescence is absent. **d-e)** Distribution of the locations of analyzed dendrite segments relative to the infarct border for control (d) and GFAP-TK+GCV mice (e) (number of segments is summed across mice, n = 8/group). **f)** Numbers of unique longitudinally tracked dendritic spines by group. Each point corresponds to an individual animal. n = 8/group. Pre-stroke spine turnover **(g)** and stability **(h)** were not different between groups ($t(14) \leq 1.1$, $p \geq 0.272$, two-tailed t tests; n = 8/group). All images are representative of experiments from 16 mice. Data are presented as mean ± SEM. Datapoints representing males are shown as circles; datapoints representing females are shown as squares. Source data are provided as a Source Data file.

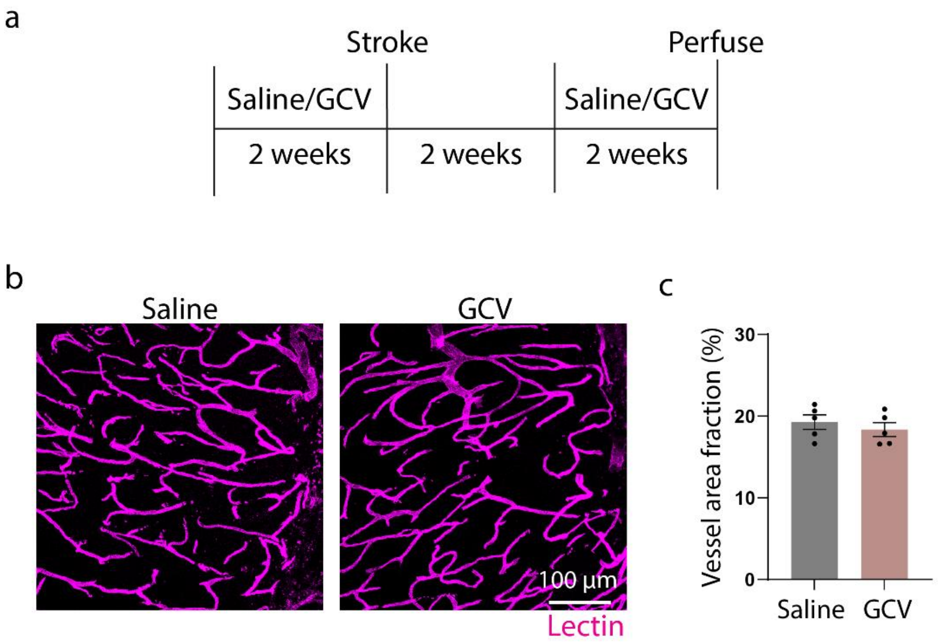

Supplementary Figure 6. Ganciclovir administration did not affect vascular remodeling after stroke.
a) Experimental design. Wildtype mice were administered saline or GCV for two weeks before stroke and again for two weeks beginning two weeks after stroke. At day 28 post-stroke, mice were injected with fluorophore-conjugated tomato lectin to label vasculature. **b)** Representative confocal images of peri-infarct vasculature. **c)** Quantification of peri-infarct vascular area fraction. $t(8) = 0.73$, $p = 0.49$, two-tailed t test (n=5/group). Source data are provided as a Source Data file.

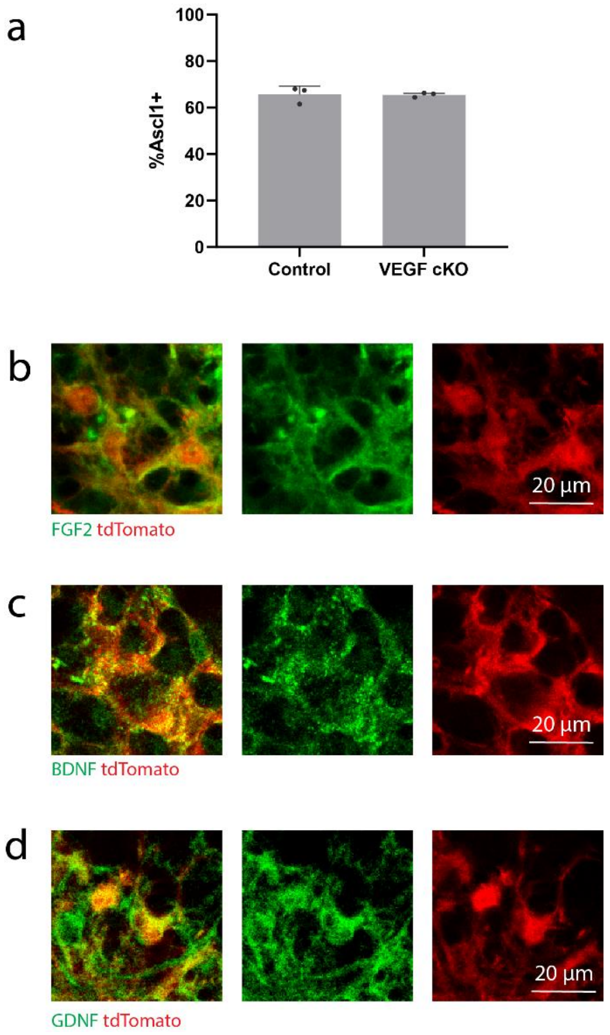

Supplementary Fig 7. VEGF cKO did not affect SVZ-derived cell differentiation or expression of other trophic factors.

a) The proportion of Ascl-expressing tdTomato$^+$ cells in peri-infarct cortex was not different between controls and VEGF cKO mice. Two-tailed t test, $t(4) = 0.06$, $p = 0.957$. $n = 3$ mice/group. **b-d)** Confocal images of VEGF cKO tdTomato$^+$ cells in peri-infarct cortex showing that expression of FGF2 (b), BDNF (c), and GDNF (d) is present in VEGF cKO mice. All images are representative of 3 mice. Source data are provided as a Source Data file.

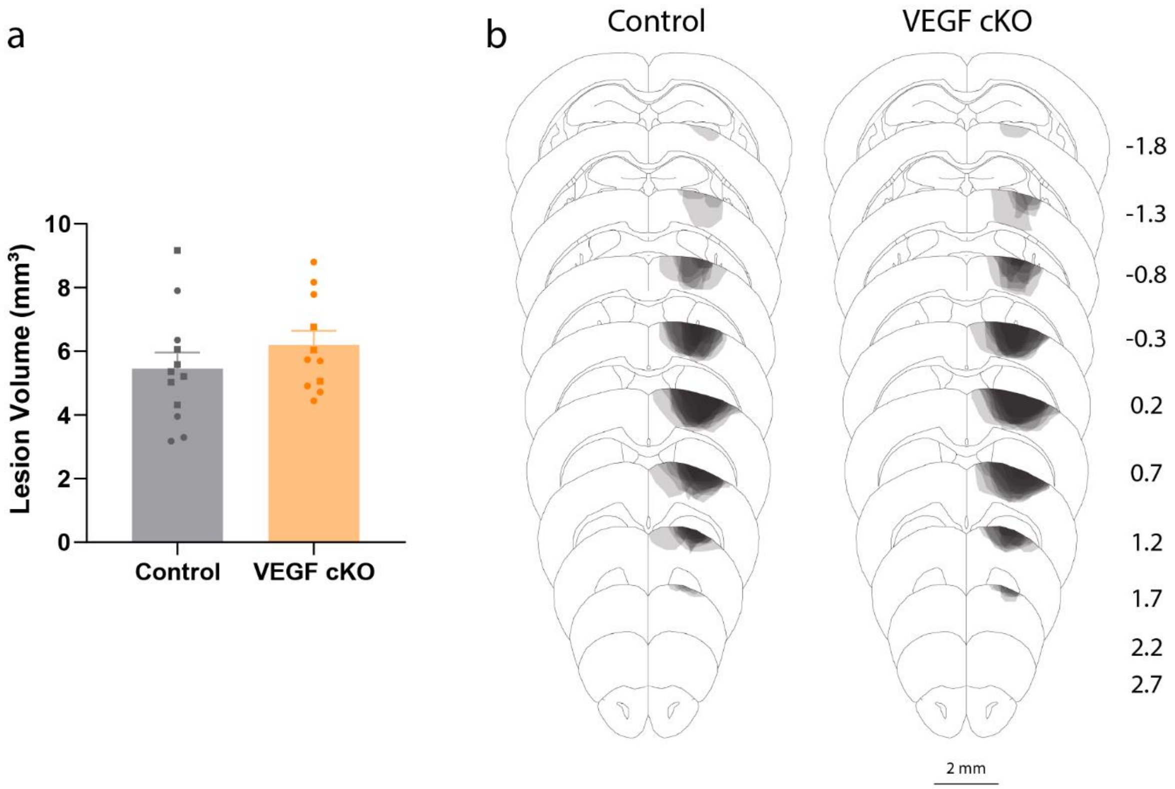

Supplementary Fig. 8. VEGF cKO did not affect lesion size or location.
a) Lesion size was not different between groups ($t(21) = 1.08$, $p = 0.292$). Two-tailed t test. n = 12 control mice, n = 11 VEGF cKO mice. **b)** Lesion reconstruction. Darker shades indicate greater overlap between animals. Source data are provided as a Source Data file.

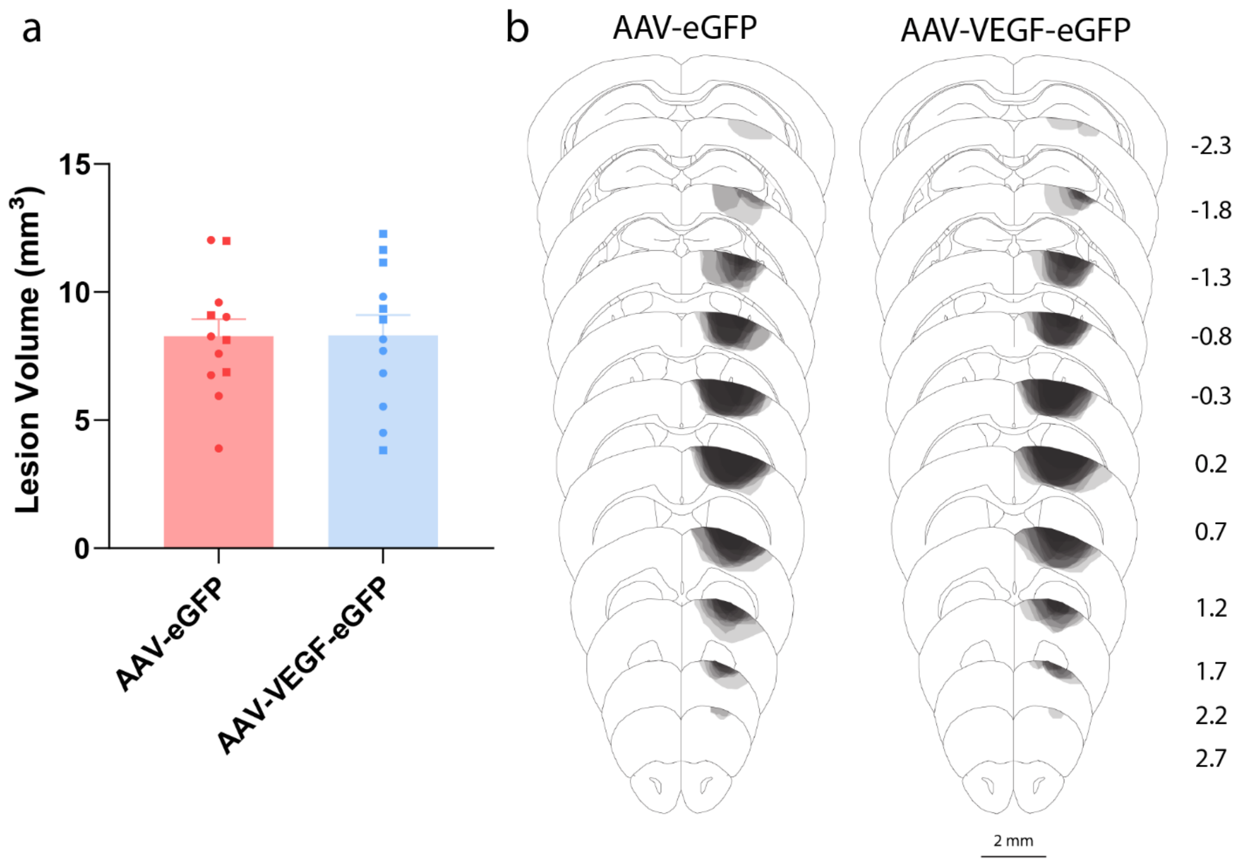

Supplementary Fig. 9. AAV-EF1α-VEGF-eGFP did not affect lesion size or location.
a) Lesion size was not different between groups ($t(22) = 0.04$, $p = 0.967$). Two-tailed t test. n = 12 mice/group. **b)** Lesion reconstruction. Darker shades indicate greater overlap between animals. Source data are provided as a Source Data file.

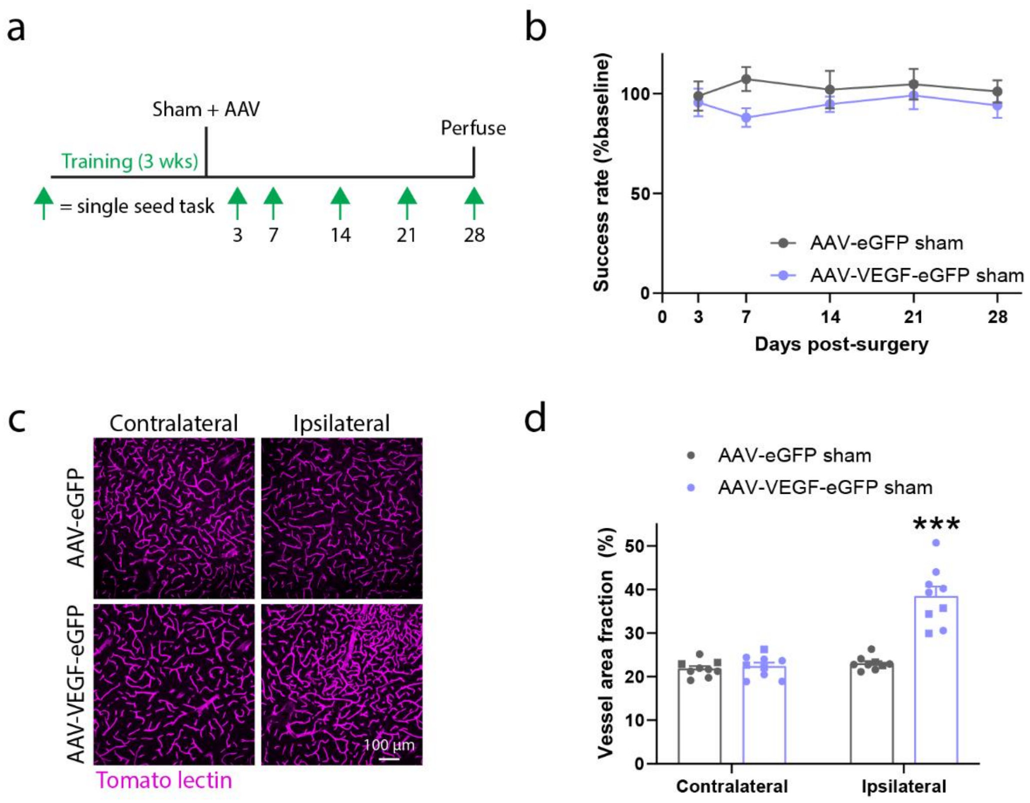

Supplementary Fig. 10. AAV- EF1α-VEGF-eGFP does not affect motor function in sham-operated animals.

a) Experimental timeline. Wildtype mice were trained on the single seed reaching task, subjected to a sham stroke procedure, and injected with either AAV-eGFP or AAV-VEGF-eGFP. Performance on the single seed task was periodically tested up to 28 days post-surgery. **b)** Performance on the single seed task was not different between groups (Two-way ANOVA, $F(1, 14) = 3.8$, $p = 0.071$, n = 8 mice/group). **c, d)** Representative images (c) and quantification (d) of vasculature in contralateral and ipsilateral cortex (relative to AAV injection site). AAV-VEGF-eGFP increased vessel density in ipsilateral cortex ($t(16) = 6.8$, ***$p < 0.0001$, two-tailed t tests, n = 9/group). Data are presented as mean ± SEM. Where individual datapoints are shown, datapoints representing males are shown as circles; datapoints representing females are shown as squares. Source data are provided as a Source Data file.

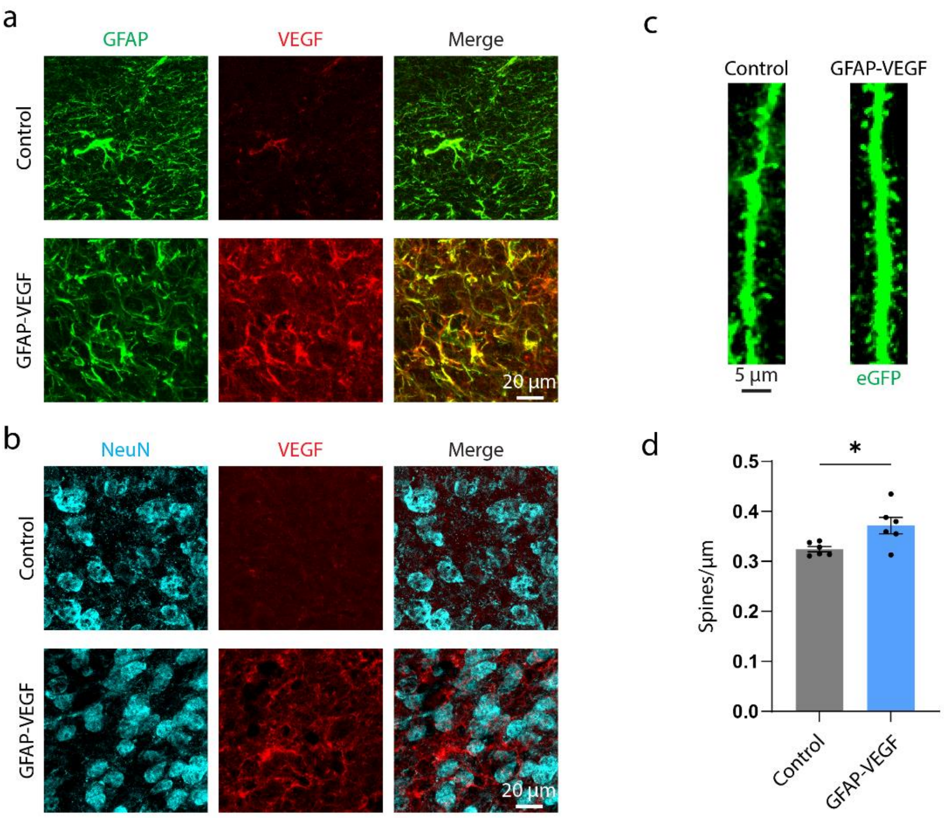

Supplementary Figure 11. VEGF overexpression in peri-infarct astrocytes enhances spine density.
a, b) Validation of AAV-GFAP-VEGF. Images are representative of experiments repeated in 3 mice per condition. a) GFAP-VEGF AAV increases expression of VEGF in astrocytes. b) GFAP-VEGF AAV does not induce VEGF expression in neurons. **c)** Representative confocal images of layer II/III dendritic spines in peri-infarct cortex of control (AAV5-CaMKIIa-eGFP+PBS) and GFAP-VEGF (AAV5-CaMKIIa-eGFP+AAV2/9-GFAP-VEGF) mice at 28 days post-stroke. **d)** Spine density was significantly higher in mice injected with AAV-GFAP-VEGF. **$t(10) = 2.7$, $p = 0.021$, two-tailed t test (n=6 mice/group). Apical dendrites were sampled from layer II/III between 100-700 µm from the infarct border. 1790 spines were counted along 5.5 mm total length of dendrite in controls (n = 6 male mice). 2015 spines were counted along 5.5 mm total length of dendrite in GFAP-VEGF mice (n = 6 male mice). Data are presented as mean ± SEM. Source data are provided as a Source Data file.
