# Supplementary material for: Subventricular zone cytogenesis provides trophic support for neural repair in a mouse model of stroke
Source: Nat Commun. 2023 Oct 10;14:6341. doi: 10.1038/s41467-023-42138-0 (PMC10564905; doi:10.1038/s41467-023-42138-0)
Supplement: Supplementary file 3 — Reporting Summary [file 41467_2023_42138_MOESM3_ESM.pdf]

nature portfolio

Corresponding author(s): Michael Williamson

Last updated by author(s): Aug 26, 2023

Reporting Summary

Nature Portfolio wishes to improve the reproducibility of the work that we publish. This form provides structure for consistency and transparency in reporting. For further information on Nature Portfolio policies, see our Editorial Policies and the Editorial Policy Checklist.

Statistics

For all statistical analyses, confirm that the following items are present in the figure legend, table legend, main text, or Methods section.

n/a	Confirmed	
☐	☒	The exact sample size (*n*) for each experimental group/condition, given as a discrete number and unit of measurement
☐	☒	A statement on whether measurements were taken from distinct samples or whether the same sample was measured repeatedly
☐	☒	The statistical test(s) used AND whether they are one- or two-sided<br>*Only common tests should be described solely by name; describe more complex techniques in the Methods section.*
☒	☐	A description of all covariates tested
☐	☒	A description of any assumptions or corrections, such as tests of normality and adjustment for multiple comparisons
☐	☒	A full description of the statistical parameters including central tendency (e.g. means) or other basic estimates (e.g. regression coefficient) AND variation (e.g. standard deviation) or associated estimates of uncertainty (e.g. confidence intervals)
☐	☒	For null hypothesis testing, the test statistic (e.g. *F*, *t*, *r*) with confidence intervals, effect sizes, degrees of freedom and *P* value noted<br>*Give P values as exact values whenever suitable.*
☒	☐	For Bayesian analysis, information on the choice of priors and Markov chain Monte Carlo settings
☒	☐	For hierarchical and complex designs, identification of the appropriate level for tests and full reporting of outcomes
☒	☐	Estimates of effect sizes (e.g. Cohen's *d*, Pearson's *r*), indicating how they were calculated

Our web collection on statistics for biologists contains articles on many of the points above.

Software and code

Policy information about availability of computer code

Data collection	The following software was used to collect data in this study:<br>Leica LAS (2.7.3.9723) was used for collection of confocal images.<br>Prairie View (5.3) was used for collection of 2-photon images.<br>Matlab (R2018a) was used for collection of MESI data.
Data analysis	The following software was used to analyze data in this study:<br>ImageJ (1.51j8) was used for image analysis.<br>GraphPad Prism (9.3) was use for statistical analysis.<br>MATLAB (R2018a) was used for analysis of MESI data.

For manuscripts utilizing custom algorithms or software that are central to the research but not yet described in published literature, software must be made available to editors and reviewers. We strongly encourage code deposition in a community repository (e.g. GitHub). See the Nature Portfolio guidelines for submitting code & software for further information.

Data

Policy information about availability of data

All manuscripts must include a data availability statement. This statement should provide the following information, where applicable:

- Accession codes, unique identifiers, or web links for publicly available datasets
- A description of any restrictions on data availability
- For clinical datasets or third party data, please ensure that the statement adheres to our policy

Source data are provided with this paper. Additional data supporting the findings of this study are provided in the Supplementary Information.

Research involving human participants, their data, or biological material

Policy information about studies with human participants or human data. See also policy information about sex, gender (identity/presentation), and sexual orientation and race, ethnicity and racism.

Reporting on sex and gender	This study did not include human participants.
Reporting on race, ethnicity, or other socially relevant groupings	This study did not include human participants.
Population characteristics	This study did not include human participants.
Recruitment	This study did not include human participants.
Ethics oversight	This study did not include human participants.

Note that full information on the approval of the study protocol must also be provided in the manuscript.

Field-specific reporting

Please select the one below that is the best fit for your research. If you are not sure, read the appropriate sections before making your selection.

[x] Life sciences [ ] Behavioural & social sciences [ ] Ecological, evolutionary & environmental sciences

For a reference copy of the document with all sections, see nature.com/documents/nr-reporting-summary-flat.pdf

Life sciences study design

All studies must disclose on these points even when the disclosure is negative.

Sample size	Sample sizes were based on past work using similar methods (Benner et al., 2013; Brown et al., 2007; Clark et al., 2019; Williamson et al., 2020, 2021).
Data exclusions	Animals were excluded from experiments involving behavioral testing if they failed to reach proficiency on the skilled reaching task (see Methods for details).
Replication	All measurements were made from at least 3 animals per groups. Experiments were run in 1-5 cohorts, with each cohort containing animals of all groups. All attempts at replication were successful.
Randomization	Mice were randomly assigned to groups except when entirely dependent upon genotype.
Blinding	Experimentation and analysis were done blinded to group allocation.

Reporting for specific materials, systems and methods

We require information from authors about some types of materials, experimental systems and methods used in many studies. Here, indicate whether each material, system or method listed is relevant to your study. If you are not sure if a list item applies to your research, read the appropriate section before selecting a response.

Materials & experimental systems

n/a	Involved in the study
☐	☒ Antibodies
☒	☐ Eukaryotic cell lines
☒	☐ Palaeontology and archaeology
☐	☒ Animals and other organisms
☒	☐ Clinical data
☒	☐ Dual use research of concern
☒	☐ Plants

Methods

n/a	Involved in the study
☒	☐ ChIP-seq
☒	☐ Flow cytometry
☒	☐ MRI-based neuroimaging

Antibodies

Antibodies used

Primary antibodies (and dilutions) used were:
Rabbit polyclonal anti-ASCL1 (1:1000) Abcam ab74065
Rabbit polyclonal anti-ASCL1 (1:500) Cosmo Bio CAC-SK-T01-003
Rabbit polyclonal anti-CD133 (1:1000) Abcam ab19898
Rabbit monoclonal anti-BDNF (EPR1292) (1:1000) Abcam ab108319
Rabbit polyclonal anti-BrdU (1:500) Abcam ab152095
Rat monoclonal anti-BrdU [BU1/75 (ICR1)] (1:500) Abcam ab6326
Rabbit polyclonal anti-cleaved caspase 3 (1:500) Cell Signaling #9661
Mouse monoclonal anti-CXCL12 (Clone # 79018) (1:200) R&D Systems MAB350
Rabbit monoclonal anti-CXCR4 (UMB2) (1:250) Abcam Ab124824
Goat polyclonal anti-DCX (1:500) Santa Cruz Biotech. Sc-8066
Rabbit monoclonal anti-ERG (EPR3864) (1:500) Abcam ab92513
Rabbit polyclonal anti-FGF2 (1:500) Sigma F-3393
Rabbit polyclonal anti-GDNF (1:100) Abcam ab18956
Rabbit polyclonal anti-GFAP (1:1000) Dako Z0334
Chicken polyclonal anti-GFAP (1:2000) Abcam ab4674
Chicken polyclonal anti-GFP (1:5000) GeneTex GTX13970
Goat polyclonal anti-HSV thymidine kinase (1:1000) Santa Cruz Biotech. Sc-28038
Rabbit monoclonal anti-Id2 (1:1000) (Clone 9-2-8) CalBioreagents M213
Rabbit polyclonal anti-Ki67 (1:500) Abcam ab66155
Mouse monoclonal anti-Nestin (Clone Rat401 (RUO)) (1:100) BD Pharmingen 556309
Mouse monoclonal anti-NeuN (clone A60) (1:500) Millipore MAB377
Rabbit monoclonal anti-NeuN (clone 27-4) (1:2000) Millipore MABN140
Rabbit polyclonal anti-Olig2 (1:1000) Millipore AB9610
Rabbit monoclonal anti-S100β (EP1576Y) (1:1000) Abcam ab52642
Rabbit polyclonal anti-Sox2 (1:1000) Millipore AB5603
Rabbit polyclonal anti-VEGF (1:1000) Millipore ABS82
Rabbit polyclonal anti-VEGF AF647 conjugate (1:1000) Millipore ABS82-AF647
Rabbit polyclonal anti-VEGF (1:250) Sigma 07-1420

Secondary antibodies (and dilutions) used were:
Alexa Fluor 488-conjugated donkey anti-chicken (1:500) Jackson ImmunoResearch 703-545-155
Alexa Fluor 488-conjugated donkey anti-goat (1:500) Jackson ImmunoResearch 705-545-147
Alexa Fluor 594-conjugated donkey anti-goat (1:500) Jackson ImmunoResearch 705-585-147
Alexa Fluor 488-conjugated donkey anti-mouse (1:500) Jackson ImmunoResearch 715-545-151
Alexa Fluor 647-conjugated donkey anti-mouse (1:500) Jackson ImmunoResearch 715-605-151
Alexa Fluor 488-conjugated donkey anti-rabbit (1:500) Jackson ImmunoResearch 711-545-152
Alexa Fluor 594-conjugated donkey anti-rabbit (1:500) Jackson ImmunoResearch 711-585-152
Alexa Fluor 647-conjugated donkey anti-rabbit (1:500) Jackson ImmunoResearch 711-605-152
Alexa Fluor 488-conjugated donkey anti-rat (1:500) Jackson ImmunoResearch 712-545-153
Alexa Fluor 594-conjugated donkey anti-rat (1:500) Jackson ImmunoResearch 712-585-153

Validation

Antibodies were validated by manufacturers and used in previously published work. Appropriate primary antibody dilutions were determined by testing multiple dilutions on sample mouse brain tissue.
Rabbit polyclonal anti-ASCL1 (Abcam ab74065): Manufacturer states that the immunogen is a synthetic peptide conjugated to KLH derived from within residues 200 to the C-terminus of Human MASH1/Achaete-scute homolog 1. The antibody was validated using mouse brain tissue. Used in PMID: 36061596.
Rabbit polyclonal anti-ASCL1 ((Cosmo Bio CAC-SK-T01-003): Manufacturer states that the immunogen is recombinant human MASH1 (ASCL1) (full length). The antibody was validated to react with mouse and human ASCL1, but not with other bHLH family proteins such as hash-2, E47, Hes-1, Hes-5, Id-3, neurogenin-1, neurogenin-2 and neurogenin-3. The antibody is validated for use in WB, IHC, IP. Used in PMID: 11736660.
Rabbit polyclonal anti-CD133 (Abcam ab19898): Manufacturer states that the immunogen is a synthetic peptide corresponding to Human CD133 (C terminal) conjugated to keyhole limpet haemocyanin. This antibody is suitable for: ICC, ICC/IF, Flow Cyt, IHC-P, IHC-

Fr, IP, WB. Validated with Caco 2, HeLa and human embryonic stem cell lysates. Reacts with: Mouse, Rat, Human. Used in PMID: 35915262.
Rabbit monoclonal anti-BDNF (Abcam ab108319): Manufacturer states that the antibody was validated in human, rat, and mouse brain tissue by immunohistochemistry. Used in PMID: 37072935.
Rabbit polyclonal anti-BrdU (Abcam ab152095): Manufacturer states that the immunogen was a chemical/ small molecule corresponding to BrdU. Antibody was validated by immunohistochemistry in mouse tissue. Used in PMID: 33910014.
Rat monoclonal anti-BrdU (Abcam ab6326): Manufacturer states that the antibody was validated in rat and mouse tissue by immunohistochemistry. Used in PMID: 36880055.
Rabbit polyclonal anti-cleaved caspase 3 (1:500) Cell Signaling #9661. Manufacturer states Cleaved Caspase-3 (Asp175) Antibody detects endogenous levels of the large fragment (17/19 kDa) of activated caspase-3 resulting from cleavage adjacent to Asp175. Produced by immunizing animals with a synthetic peptide corresponding to amino-terminal residues adjacent to (Asp175) in human caspase-3. Used for immunohistochemistry in mouse brain tissue in PMID: 33910014.
Mouse monoclonal anti-CXCL12 (1:200) R&D Systems MAB350: Manufacturer states that the immunogen was E. coli-derived recombinant human CXCL12/SDF-1 alpha Lys22-Lys89. The antibody reacts with human and mouse CXCL12. Validated for immunohistochemistry in human tonsil tissue. Used for immunohistochemistry in PMID: 33602937.
Rabbit monoclonal anti-CXCR4 (1:250) Abcam Ab124824: Manufacturer states that the immunogen was a synthetic peptide within Human CXCR4 aa 300 to the C-terminus. Validated in the following conditions: B: HeLa, Jurkat and WI-38 cell lysates; HEK239 transfected with CXCR4, cell lysate. ICC/IF: Jurkat cells. IHC-P: Human cervical carcinoma, bladder cancer tissue, ovarian adenocarcinoma tissue and tonsil tissue. Flow Cyt (intra): Jurkat cells, HEK-293T cells transfected with human CXCR4 expressed vector. IHC-Fr: Mouse and rat E14.5 cerebrum. Used in PMID: 37532885.
Goat polyclonal anti-DCX (1:500) Santa Cruz Biotech. Sc-8066: Manufacturer states epitope mapping at the C-terminus of Doublecortin of human origin. Used for immunohistochemistry in mouse brain tissue in PMID: 26337870.
Rabbit monoclonal anti-ERG (1:500) Abcam ab92513: Manufacturer states validated in WB: Jurkat, HeLa and RAW 264.7 cell lysates; Rat brain and heart lysates. IHC-P: Human kidney, brain and prostate adenocarcinoma tissues; Fus A5 transgenic mouse prostate tissue; Mouse brain tissue. ICC/IF: Circulating tumor cells (CTCs) from a castrate-resistant prostate cancer (CRPC) patient; THP-1 cells. Flow Cyt (intra): THP-1 cells. Immunogen is a synthetic peptide within Human ERG aa 450 to the C-terminus. Used in PMID: 37537199.
Rabbit polyclonal anti-FGF2 (1:500) Sigma F-3393: Manufacturer states immunogen is a synthetic peptide sequence corresponding to bovine FGF2 (1-24). Validated in immunohistochemistry. Used in PMID: 32344828.
Rabbit polyclonal anti-GDNF (1:100) Abcam ab18956: Manufacturer states immunogen is a synthetic peptide surrounding amino acid 195 of mouse GDNF. Used for immunohistochemistry in CNS tissue in PMID: 31111776.
Rabbit polyclonal anti-GFAP (1:1000) Dako Z0334: Manufacturer states immunogen is GFAP isolated from cow spinal cord. The antibody has been solid-phase absorbed with human and cow serum proteins. In crossed immunoelectrophoresis using 50 µL antibody per cm2 gel area, no reaction with 2 µL human plasma and 2 µL cow serum is observed. The antibody shows one distinct precipitate (GFAP) with cow brain extract. GFAP shows 90-95% homology between species, and, as demonstrated by immunohistochemistry, the antibody reacts strongly with human GFAP. Used for immunohistochemistry in mouse brain tissue in PMID: 33910014.
Chicken polyclonal anti-GFAP (1:2000) Abcam ab4674: Manufacturer states immunogen is recombinant full length protein corresponding to Human GFAP. Isotype 1 expressed in and purified from E. coli. Validated for immunohistochemistry in mouse brain tissue. Used in PMID: 37393623.
Chicken polyclonal anti-GFP (1:5000) GeneTex GTX13970: Manufacturer states immunogen is purified recombinant green fluorescent protein (GFP) emulsified in Freund's adjuvant. Purified by antigen-affinity chromatography. Validated for immunohistochemistry in mouse brain tissue. Used in PMID: 36224417.
Goat polyclonal anti-HSV thymidine kinase (1:1000) Santa Cruz Biotech. Sc-28038. Manufacturer states immunogen is a peptide mapping within an internal region of HSV-1 Thymidine Kinase. Used for immunohistochemistry in mouse brain tissue in PMID: 33910014.
Rabbit monoclonal anti-Id2 (1:1000) CalBioreagents M213: Validated for immunohistochemistry in human mammary tumor. Reacts with mouse and human Id2. Used for immunohistochemistry in PMID: 29103804.
Rabbit polyclonal anti-Ki67 (1:500) Abcam ab66155: Manufacturer states the antibody is knockout validated and demonstrates its use for immunohistochemistry in mouse brain tissue. Used in PMID: 36695896.
Mouse monoclonal anti-Nestin (1:100) BD Pharmingen 556309: Manufacturer states immunogen is Rat (E15) spinal cord extracts. Used for immunohistochemistry in mouse brain tissue in PMID: 35912073.
Mouse monoclonal anti-NeuN (1:500) Millipore MAB377: Manufacturer states validated for use in the following: FC, IC, IF, IH, IH(P), IP and WB. Used for immunohistochemistry in mouse brain tissue in PMID: 37288657.
Rabbit monoclonal anti-NeuN (1:2000) Millipore MABN140: Manufacturer states validated for use in immunohistochemistry in mouse brain tissue. Used in PMID: 35177618.
Rabbit polyclonal anti-Olig2 (1:1000) Millipore AB9610: Manufacturer confirms the antibody was used for immunostaining on human, rat and mouse cell lines (human oligodendroglioma, rat primary neurepithelial, mouse transfected) and tissues (brain and spinal cord). Used in PMID: 37046092.
Rabbit monoclonal anti-S100β (1:1000) Abcam ab52642: Manufacturer states immunogen is a synthetic peptide within Human S100 beta aa 50 to the C-terminus. Validated for immunohistochemistry in human, mouse and rat cerebral cortex. Used for immunohistochemistry in mouse brain tissue in PMID: 33910014.
Rabbit polyclonal anti-Sox2 (1:1000) Millipore AB5603: Manufacturer states immunogen is a KLH-conjugated linear peptide corresponding to a C-terminal region sequence of human Sox2. Used for immunohistochemistry in mouse brain tissue in PMID: 36535938.
Rabbit polyclonal anti-VEGF (1:1000) Millipore ABS82: Manufacturer states immunogen is a KLH-conjugated linear peptide corresponding to human VEGF at the N-terminus. Knockout validated for immunohistochemistry in mouse brain tissue in this article.
Rabbit polyclonal anti-VEGF AF647 conjugate (1:1000) Millipore ABS82-AF647: Manufacturer states immunogen is a KLH-conjugated linear peptide corresponding to human VEGF at the N-terminus. Knockout validated for immunohistochemistry in mouse brain tissue in this article.
Rabbit polyclonal anti-VEGF (1:250) Sigma 07-1420: Manufacturer states immunogen is highly pure (>98%) recombinant mouse Vascular Endothelial Growth Factor. Validated for use in WB, IH(P), ELISA. Used for immunohistochemistry in mouse tissue in PMID: 26195726.

All secondary antibodies (Jackson Immunoresearch) were purified from antisera by immunoaffinity chromatography using antigens coupled to agarose beads. These antibodies display minimal cross reactivity to off-target species.

Animals and other research organisms

Policy information about studies involving animals; ARRIVE guidelines recommended for reporting animal research, and Sex and Gender in Research

Laboratory animals	Mice (3-16 months old) used were:<br>Ai14: B6.Cg-Gt(ROSA)26Sortm14(CAG-tdTomato)Hze/J, The Jackson Laboratory JAX #007914<br>GFAP-TK: B6.Cg-Tg(Gfap-TK)7.1Mvs/J, The Jackson Laboratory JAX #005698<br>Nestin-CreER: C57BL/6-Tg(Nes-cre/ERT2)KEisc/J, The Jackson Laboratory JAX #016261<br>ASCL1-CreER: Ascl1tm1.1(Cre/ERT2)Jejo/J, The Jackson Laboratory JAX #012882<br>CAG-LSL-Sun1-sfGFP: B6;129-Gt(ROSA)26Sortm5(CAG-Sun1/sfGFP)Nat/J, The Jackson Laboratory JAX #021039<br>Thy1-GFP: Tg(Thy1-EGFP)MJrs/J, The Jackson Laboratory JAX #007788<br>Floxed Vegfa, Genentech, Gerber et al. 1999
Wild animals	This study did not use wild animals.
Reporting on sex	Mice of both sexes were used for all experiments. Data from each sex is shown with distinct data points (see figure legends).
Field-collected samples	This study did not involve field-collected samples.
Ethics oversight	Animal use was approved (AUP-2018-00283) by the Institutional Animal Care and Use Committees at the University of Texas at Austin or Baylor College of Medicine.

Note that full information on the approval of the study protocol must also be provided in the manuscript.
