# Supplementary material for: Subventricular zone cytogenesis provides trophic support for neural repair in a mouse model of stroke
Source: Nat Commun. 2023 Oct 10;14:6341. doi: 10.1038/s41467-023-42138-0 (PMC10564905; doi:10.1038/s41467-023-42138-0)
Supplement: Supplementary file 4 — Description of Additional Supplementary Files [file 41467_2023_42138_MOESM4_ESM.pdf]

Description of Additional Supplementary Files

Supplementary Data 1: List of reagents.
